# Supplementary material for: The Effects of Aflatoxin B1 on Liver Cholestasis and Its Nutritional Regulation in Ducks
Source: Toxins (Basel). 2024 May 24;16(6):239. doi: 10.3390/toxins16060239 (PMC11209606; doi:10.3390/toxins16060239)
Supplement: Supplementary file 1 [file toxins-16-00239-s001.zip › toxins-3007293-supplementary.pdf]

toxins

The Effects of Aflatoxin B_1 on Liver Cholestasis and Its Nutritional Regulation in Ducks

Aimei Yu, Huanbin Wang, Qianhui Cheng, Shahid Ali Rajput and Desheng Qi

Table S1. Composition of basal diets and nutrient levels [1].

Ingredients	Percentage (%)	Nutrient level	Content
corn	65.50	ME (kcal/kg)	2800
Soybean meal	30.30	Crude protein (%)	19.00
Soybean oil	1.00	Calcium (%)	0.80
Limestone	1.20	Available phosphorus (%)	0.28
salt	0.3	Met (%)	0.50
Choline chloride	0.1	Lys (%)	1.12
Lys	0.36	Met + Cys (%)	0.85
Met	0.24		
Premix[1]	1.00		

[1]The premix provided the following per kg of diet: vitamin A, 10,000 IU; cholecalciferol 2,500 IU; vitamin E, 35 mg; thiamine, 2.50 mg; riboflavin, 10 mg; pyridoxine, 5.5 mg; iron, 80 mg; manganese, 90 mg; copper, 10 mg; zinc, 60 mg; selenium, 0.4 mg; iodine, 0.3 mg.

Table S2. Primers for PCR.

Gene name	Gene ID	Primer sequence (5'-3')	Product size (bp)
CYP7A1	101790267	F: CAAAGCAGGAGACCGAGAGA<br>R: CATTCAGGAACATGCGGAGG	216
CYP27A1	118169577	F: CAACCTGCTCTATCGCTTCG<br>R: CCAGCTCCTTCATCTTCCGA	263
CYP8B1	101797490	F: CAACATCGTCTTCAGAGCCG<br>R: CCGCTCAGCTTCAAGTTTGT	199
BSEP	101797164	F: GTGTTGCTTGTGTTTGGTGC<br>R: TTGTTCAATGTCCAGCAGCC	177
SHP	101791271	F: TTTCGTCTTGTCCGTCCTGA<br>R: CTGTTTCGATGTCAGCAGC	279
OATP	101802312	F: AAGCAGCATTTCACCACTGG<br>R: GGCATGATGGGAGTTTCACC	183
GAPDH	101803965	F: TGAAAGTCGGAGTCAACGGA<br>R: CATCTGATGTTGCTGGGGTC	249
